# Application of Artificial Neural Networks to the Analysis of Friction Behaviour in a Drawbead Profile in Sheet Metal Forming

**DOI:** 10.3390/ma15249022

**Published:** 2022-12-16

**Authors:** Tomasz Trzepieciński, Sherwan Mohammed Najm

**Affiliations:** 1 Department of Manufacturing Processes and Production Engineering, Faculty of Mechanical Engineering and Aeronautics, Rzeszow University of Technology, Al. Powst. Warszawy 8, 35-959 Rzeszów, Poland; 2 Department of Manufacturing Science and Engineering, Budapest University of Technology and Economics, Műegyetemrkp 3, H-1111 Budapest, Hungary; 3 Kirkuk Technical Institute, Northern Technical University, Kirkuk 36001, Iraq

**Keywords:** coefficient of friction, friction, metal forming, surface roughness

## Abstract

Drawbeads are used when forming drawpieces with complex shapes to equalise the flow resistance of a material around the perimeter of the drawpiece or to change the state of stress in certain regions of the drawpiece. This article presents a special drawbead simulator for determining the value of the coefficient of friction on the drawbead. The aim of this paper is the application of artificial neural networks (ANNs) to understand the effect of the most important parameters of the friction process (sample orientation in relation to the rolling direction of the steel sheets, surface roughness of the counter-samples and lubrication conditions) on the coefficient of friction. The intention was to build a database for training ANNs. The friction coefficient was determined for low-carbon steel sheets with various drawability indices: drawing quality DQ, deep-drawing quality DDQ and extra deep-drawing quality EDDQ. Equivalents of the sheets tested in EN standards are DC01 (DQ), DC03 (DDQ) and DC04 (EDDQ). The tests were carried out under the conditions of dry friction and the sheet surface was lubricated with machine oil LAN46 and hydraulic oil LHL32, commonly used in sheet metal forming. Moreover, various specimen orientations (0° and 90°) in relation to the rolling direction of the steel sheets were investigated. Moreover, a wide range of surface roughness values of the counter-samples (Ra = 0.32 μm, 0.63 μm, 1.25 μm and 2.5 μm) were also considered. In general, the value of the coefficient of friction increased with increasing surface roughness of the counter-samples. In the case of LAN46 machine oil, the effectiveness of lubrication decreased with increasing mean roughness of the counter-samples Ra = 0.32–1.25 μm. With increasing drawing quality of the sheet metal, the effectiveness of lubrication increased, but only in the range of surface roughness of the counter-samples in which Ra = 0.32–1.25 μm. This study investigated different transfer functions and training algorithms to develop the best artificial neural network structure. Backpropagation in an MLP structure was used to build the structure. In addition, the COF was calculated using a parameter-based analytical equation. Garson partitioning weight was used to calculate the relative importance (RI) effect on coefficient of friction. The Bayesian regularization backpropagation (BRB)—Trainbr training algorithm, together with the radial basis normalized—Radbasn transfer function, scored best in predicting the coefficient of friction with R^2^ values between 0.9318 and 0.9180 for the training and testing datasets, respectively.

## 1. Introduction

Sheet metal forming (SMF) includes a number of various plastic working processes carried out mainly in cold-forming conditions and used to form semi-finished products in the form of sheet metals. Deep drawing is carried out with devices called dies, usually mounted on hydraulic or mechanical presses. There are methods of unconventional forming involving shaping with a rotating tool [1,2,3]. During deep drawing, a flat blank is transformed into a three-dimensional drawpiece with a non-expandable surface [4,5]. When forming non-axisymmetric parts, especially in the automotive industry, the dies usually require drawbeads.

The drawbead used during the SMF process is used to limit the flow of material in certain areas of the drawpiece. This control is obtained using a restraining force supplied either by the drawbeads and/or a blankholder tool [6]. The sheet curvature changes many times during the passage of the sheet metal through the drawbead. The sheet material is also plastically deformed and subjected to the effects of friction. The friction conditions existing on the drawbead determine the final quality of the product’s surface and the required height of the drawbead to obtain the appropriate restraining force. The drawbeads play a key role in sheet metal forming and its function becomes indispensable for the forming of drawpieces with complicated shapes [7].

Friction in sheet-metal-forming processes is a complex function of tool- and sheet-surface roughness, friction conditions, lubricant properties, sliding velocity and pressure values [8,9,10]. In most SMF processes, the occurrence of friction is an undesirable phenomenon and causes [11,12,13,14] increased forming force, irregular deformation, and a reduction in product quality and tool life. The main parameters influencing tribological phenomena in SMF also include the dynamics of load, kinematics of tool movement, physicochemical phenomena occurring on the contact surface of the tool and workpiece and, finally, the temperature of the forming process [7,15,16]. The most effective way to reduce the unfavourable effects of the frictional forces on the quality of the product and the course of the forming process is the use of lubrication with the use of solid lubricants, oils or emulsions [17]. A number of tribological tests have been developed to model the friction phenomena in specific areas of the forming die, i.e., strip drawing test [18,19], bending under tension tests [20,21] and drawbead simulator tests [12]. The change in the friction conditions in the drawbead simulator test analysed in this paper was obtained by changing the contact angle of the counter-sample [22,23], the shape and dimensions of the thrust threshold model [24,25], the lubrication conditions (dry friction or lubrication) [22,24,26] and the speed of sheet metal drawing [24,26,27].

In recent years, many authors have considered the effects of the blank holding force and the drawbead on the SMF processes. Huh et al. [28] analysed the quantitative effect of the drawbead on the SMFed part. To overcome wrinkling caused by non-uniform metal flow, the authors proposed forming analyses with semi-open- and open-type channels. It was found that forming analysis with semi-open-type channels was more appropriate than that with open-type channels. Dahham et al. [29] proposed an approach to control the final shape of the workpiece after elastic springback when using a drawbead. They also numerically predicted the thinning failure and wrinkling when forming cylindrical drawpieces. A half circle shape of drawbead was found to be the optimal solution with regard to thickening and thinning of the drawpiece wall. Wu et al. [30] analysed the effect of strain-induced surface changes on wear in strip drawing tests with drawbead geometry. They found that the strain difference between both sides of the sheet strip had a minor effect on wear behaviour. Bassoli et al. [31] developed an experimental approach to measure the restraining force exerted on sheet metal by means of a versatile drawbead simulator. The authors found that the normalised restraining force, which is the restraining force normalised by drawbead width, increased with increasing values of the bead height-to-radius ratio. Quenching the drawbead material reduced the effectiveness of the restraining action due to a reduction in the coefficient of friction. Manjula et al. [32] observed that the average contact angle increased with the depth of drawbead penetration and that the actual coefficient of friction was a function of the contact angle. Jansson et al. [33] optimised the draw-in for non-axisymmetric auto-body parts by using a try-out tool for a part that was defined by CAD-data surfaces perfectly corresponding to the actual tool surfaces. However, the method proposed implied producing tools with different drawbeads every time when a new shape of the drawpiece has to be validated. Figueiredo et al. [26] used the drawbead test to analyse the evolution of the coefficient of friction with increasing surface roughness of the die. It was concluded that the surface roughness of the die material had a significant effect on the coefficient of friction. Duarte et al. [6] proposed a method that was developed using the similitude approach in order to understand the effect of the most important parameters on the drawbead restraining force. The results of the explicit finite element method (FEM) were compared with the analytical model of Stoughton [34] and experimental results provided by Nine [35] (the maximum discrepancy was found to be below 11%). Nanayakkara et al. [36] developed formulae for the determination of the coefficient of friction for partial penetration of the drawbead. Cillaurren et al. [37] statistically analysed the sliding velocity and contact pressure ranges in sheet metal forming processes. The contact pressure and sliding velocity corresponding to a drawbead region were numerically analysed though FE-based numerical modelling. Duarte and Oliveira [38] analysed the influence of sheet thickness on the drawbead restraining force in SMF. It was found that the drawbead restraining force increased linearly with respect to sheet thickness. Billade and Dahake [39] designed drawbeads in such a way that they produced a part with a thickness of less than 20%, i.e., the desired value. It was concluded that circular drawbeads were preferred because thinning was less significant with the circular bead compared to the step bead. Samuel [22] investigated the behaviour of metal flow passing through two shapes of drawbeads, i.e., single square and single circular female. The results of the FEM were in good agreement with the experimental ones. Chen et al. [40] presented an experimental study focused on the drawbead restraining force and friction characteristics of dual-phase steels. The influence of surface roughness, material properties, sliding velocity, temperature, and lubricant and absence of it on friction behaviour was studied. It was found that lubrication decreased the COF at room temperature and increased the friction coefficient at elevated temperatures. Furthermore, the drawbead restraining force was increased with the decrease in drawbead radius. Weinmann et al. [41] developed a multiple-action hydraulic drawbead simulator for the purpose of studying the effectiveness of feedback controls in SMF. It was concluded that a pre-specified trajectory of drawbead restraining force can be followed in spite of changing friction conditions in the drawbead. The experimental tests also indicated the effectiveness of feedback control in reducing the sensitivity of the system to process parameter variations and external disturbances.

Recently, various techniques of artificial intelligence (IE), i.e., genetic algorithms, fuzzy systems, artificial neural networks, and cognitive systems, have been used in the metal forming industry. Machine learning (ML) has been applied using various artificial neural network techniques in a number of applications in tribology [42,43]. Two main methods in supervised machine learning are regression and classification. In the field of tribology, many tests on materials are typically performed, which define a set of tribological properties [43]. Machine learning systems easily identify trends in data and exhibit the ability to learn. It allows them make predictions and improve the algorithms on their own. ML systems are good at handling data that are multi-variety and multi-dimensional. ML algorithms requires massive datasets to train on, which is the main disadvantage. Machine learning is autonomous but highly susceptible to errors in processing small datasets [44]. They also need massive computational resources to function in the case of big data analyses. Among the applications of machine learning models, the most recent are the ensemble methods [45], kernel methods [46], linear methods [47] and the artificial neural network methodology [48]. In the literature, machine learning algorithms have already been applied to a variety of manufacturing issues, such as estimating tool wear in milling operations [49], predicting tool life in micro-milling [50], the estimation of energy consumption in metal-forming processes [51] and analysis of bending processes [52].

The applications of AI that have seen the most progress during the past two decades are diverse, including deep learning in sheet metal bending [53], brake performance [54,55], wheel and rail wear [56], energy estimation consumption in metal forming [51], buckling instability prediction [57], extra deep drawing (EDD) steel surface roughness [58], and tool wear [59], just a few areas where tribology research has been steadily growing. ANNs are soft computing tools that can build linear and non-linear models [60]. The network’s structure and operation resemble the brain and comprise linked neurons that process information in parallel. ANN architecture contains input, output and hidden layers. Most analyses require one hidden layer [61]. After training, ANNs may predict output signals based on input and output signals.

Lee et al. [57] used an artificial intelligent self-learning algorithm to predict the buckling instability for the reliable design of sheet metal panels. Liu et al. [53] developed a novel theory-guided regularization method for training deep neural networks. The developed method was used to investigation of the springback phenomenon in sheet metal bending processes. Najm and Paniti [2] used a machine learning algorithm to investigate the parametric effects of single point incremental forming on the pillow effect and wall profile of AlMn1Mg1 aluminium alloy sheets. Shamsuzzohaa et al. [62] used machine learning and deep learning algorithms to detect the minimum gaps between components produced after the punching operation of metal sheet. The outcome of this study contributed to eliminating the production bottleneck. In the context of triboinformatics or tribology 4.0 [63], advanced data handling, analysis and learning methods can be developed based upon this sound and data-rich foundation [64]. There has great potential to automatize and optimize the data acquisition and processing, which is presently still very manual in the field of tribology.

The aim of this paper is application of ANNs to understand the effect of the various parameters, i.e., sheet strip orientation, surface roughness of tool (counter-samples), lubrication conditions and mechanical parameters of the workpiece material on the coefficient of friction (COF). Due to the many parameters that influence friction and determine the relationship between input and output parameters (COF), we decided to apply artificial neural networks (ANNs). To the best of the authors knowledge, an analysis of this kind has not previously been undertaken with regard to friction in the drawbead region of the stamping tool. The problem of separating the frictional resistance from the total resistance of the sheet passing through the drawbead was solved using a specially designed and manufactured drawbead simulator mounted on a universal tensile testing machine. Experiments were conducted using a special drawbead simulator for determining the value of the coefficient of friction on the drawbead. It was found that COF increased with the mean surface roughness of the counter-samples for dry and lubricated friction conditions. The multilayer perceptron was trained using experimental data with an average R^2^ value of 0.9293 for both the trained and tested datasets. The analysis of the relative significance showed that sample orientation in relation to the rolling direction of the steel sheets and strengthening coefficient significantly affect COF. The strain hardening exponent has only a slight influence on the COF.

## 2. Material and Methods

### 2.1. Test Material

Three types of deep-drawing low-carbon steel sheets were used in the research: 1 mm thick drawing quality (DQ) steel sheet, 0.8 mm thick deep drawing quality (DDQ) steel sheet and 1 mm thick extra deep drawing quality (EDDQ) steel sheet. The sheets were fabricated according to the national Polish standard PN 87/H–92143. However, according to the EN 10130:2009 standard the grade DQ corresponds to the DC01 steel sheet, grade DDQ corresponds to the DC03 steel sheet, and grade EDDQ corresponds to the DC04 steel sheet. Approximate equivalents of the DQ, DDQ and EDDQ steel sheets in AISI standards are A366, A619, A620, respectively.

**Figure 1:**
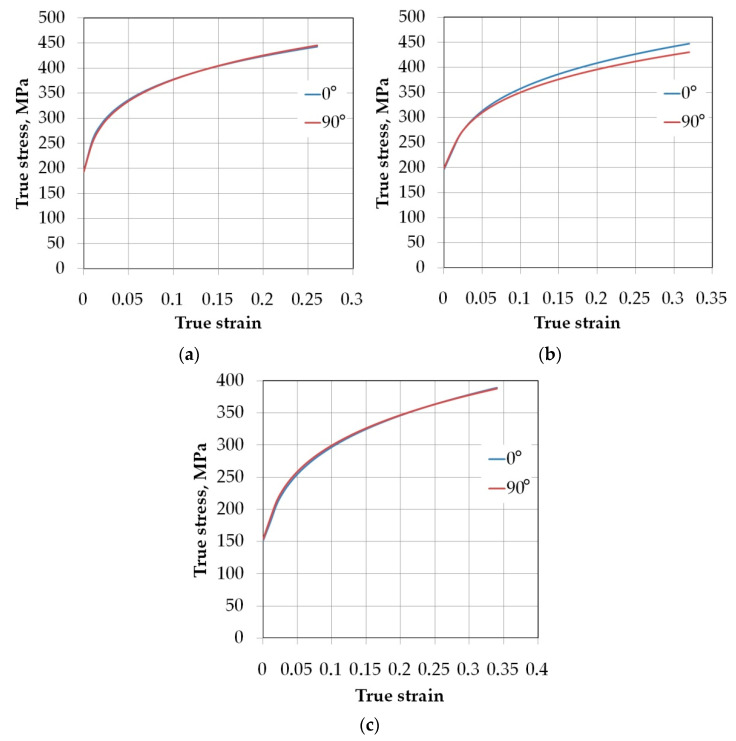
The true stress–true strain curves of the test sheets: (**a**) DQ, (**b**) DDQ and (**c**) EDDQ.

The sheets tested are commonly used in the automotive industry for the fabrication of drawpieces with complex shapes. The basic mechanical parameters of the sheet metals (Table 1) were determined in a uniaxial tensile test according to ISO 6892-1 [65] on specimens cut transverse to the rolling direction (90°) and along the rolling direction (0°). The values of the work hardening parameters (strengthening coefficient K and strain hardening exponent n) were determined based on an approximation of the true stress–true strain relation (Figure 1) by the power law equation σ_p_ = K·ε^n^, where σ_p_ is the true stress and ε is plastic strain.

**Figure 2:**
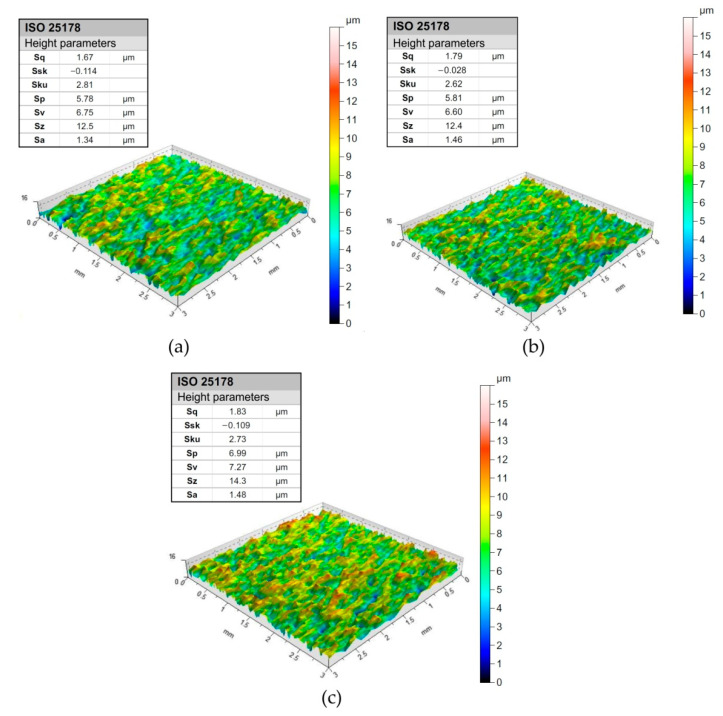
The surface topographies of the test sheets: (**a**) DQ, (**b**) DDQ and (**c**) EDDQ.

The measurement of the surface roughness parameters of the test sheets was carried out using a T8000RC profilometer in accordance with the requirements of the ISO 25178 standard [66]. The main standard 3D parameters and surface topographies determined by this measurement are listed in Figure 2. Sheet surfaces were sampled with an area of 3 mm × 3 mm.

### 2.2. Experimental Setup

**Figure 3:**
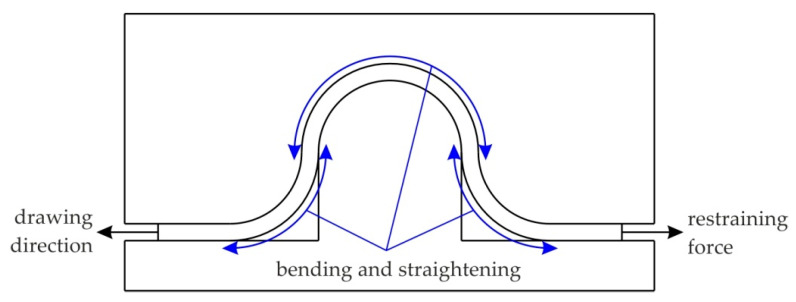
The bending and straightening of the sheet metal in the drawbead region.

**Figure 4:**
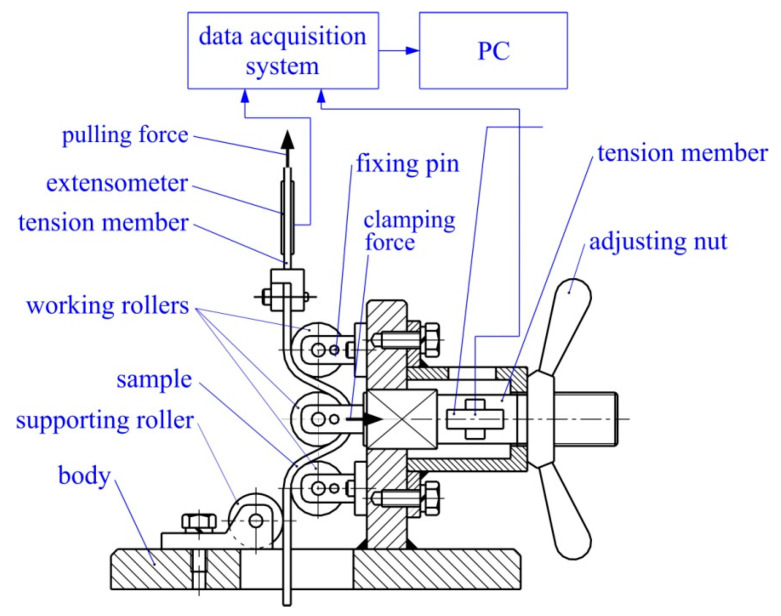
The drawbead simulator.

The drawbead test permitted the simulation of the bending and unbending in SMF and the measurement of the COF during the sliding of the sheet against a die. During the passage of the sheet metals through the drawbead, the sheet is bent and straightened three times (Figure 3). The idea behind the design of the proposed drawbead simulator (Figure 4) is the possibility of separating the frictional resistance of the sheet from the total resistance of the passage of the sheet metal through the drawbead and thus determining the value of the COF on the basis of the concept proposed by Nine [35].

The tester consists of a body to which three working counter-samples and one guide counter-sample made of X165CrV12 cold-work tool steel were attached. The tester was mounted on the lower grip of a uniaxial tensile testing machine. The rotational motion of the working counter-samples was blocked by fixing pins. The value of the drawbead height was set by holding notes. The diameter of the counter-samples was 20 mm. During the tests, the pulling force and clamping force were measured using strain gauges. Measurement signals were transmitted to the 8-channel HBM amplifier, and then to a computer with QuantumX Assistant v1.1 R1 software, enabling cooperation with the amplifier and the recording of the clamping and pulling forces.

**Figure 5:**
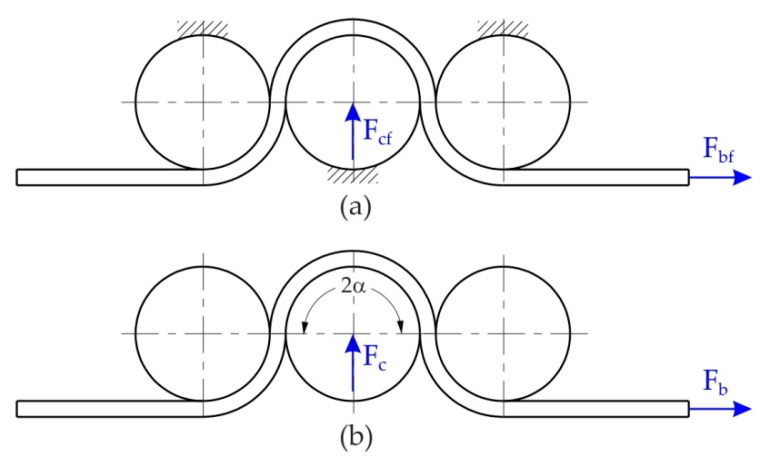
A conceptual diagram of the process for measuring force in the drawbead test in the presence of (**a**) fixed and (**b**) rotatable counter-samples.

The friction test consisted of pulling the sheet strips in conditions involving either fixed or rotatable cylindrical counter-samples. During friction with the participation of fixed counter-samples, changes were registered in the pulling force F_bf_ and the clamping force F_cf_. Similarly, under frictional conditions with freely movable rollers, the pulling force F_b_ and the clamping force F_c_ were measured (Figure 5). The friction test realised in the presence of fixed counter-samples represents the total resistance of the sheet metal passing through the drawbead. 

The value of the COF was determined according to the relationship [35]:

(1) $$\mu =\frac{\sin\alpha }{2\alpha }\cdot \frac{F_{\mathrm{bf}}-F_{b}}{F_{\mathrm{cf}}}$$

where: F_b_ is the pulling force at the rotatable rollers, F_cf_ is clamping force with fixed rollers, Fbf is pulling force with fixed rollers, and 2α = π/2 confirms a full penetration of the middle counter-sample.

Samples in the form of strips of sheet 200 mm long and 20 mm wide were used as the test material. They were cut lengthways and transversely to the rolling direction of the sheet. One end of the sample was mounted on an upper grip of a uniaxial tensile testing machine. The pulling speed of the sample was 1 mm/s. Four sets of counter-samples were used with a surface roughness Ra equal to 0.32, 0.63, 1.25 and 2.5 μm in such a way as to ensure a wide range of variability in the Ra roughness parameter. Before starting the tests, the samples were cleaned with acetone. The tests were carried out under the conditions of dry friction and lubrication of the sheet surface with machine oil LAN46 and hydraulic oil LHL32. The basic physical properties of the oils based on the material cards provided by the producers are presented in Table 2.

### 2.3. Artificial Neural Networks

Rumelhart, McClelland and Hinton introduced the multilayer perceptron (MLP) notion in 1986, after Werbos’ 1974 proposal [67]. Network topology is the layout of network elements and their inputs, outputs and connections. The structure of an ANN can be described by the number of input and output layers, transfer functions between these levels and neurons in each layer [68]. A hidden layer separates the ANN’s input and output layers. Each network layer has many neurons. The number of input variables and output neurons is the same. Based on the transfer or activation function, these layers’ neurons transfer weight backward and forward [69]. Backpropagation learning to model an ANN with a multilayer perceptron (MLP) structure was used in this study. The multilayer perceptron is defined in Equation (2):

(2) $$y=f\left(net\right)=f\left(\overset{n}{\underset{i=1}{\sum }}w_{i}x+b\right)$$

where y is the output and x is the input, $w_{i}$ are the weights and b is the bias [70].

**Figure 6:**
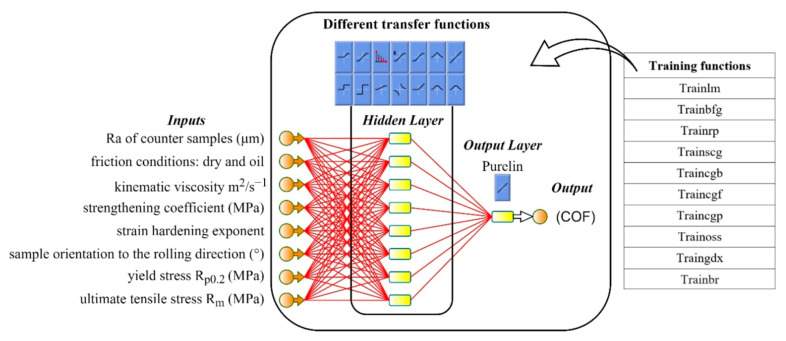
Multilayer perceptron structures.

COF values were predicted using MLP structures in MATLAB R2022a [71]. Figure 6 shows that the network structure has one hidden layer with 10 neurons linking to the input and output layers for this investigation. Target datasets utilizing real measured data from three material investigations were adopted. The whole data was accumulated from three types of deep-drawing steel sheets (DQ, DDQ, and EDDQ). Eight neurons were inputs: Ra of the counter-samples (μm), friction conditions: dry and oil, kinematic viscosity m^2^·s^−1^, sample orientation to the rolling direction (°), yield stress R_p0_._2_ (MPa), ultimate tensile stress R_m_ (MPa), strengthening coefficient (MPa), strain hardening exponent, and COF values were outputs. It is worth to mention that two oils were chosen: machine oil LAN46 and hydraulic oil LHL32. This study employed 1000 epochs, 0.01 and 0.0001 as training parameters. 

**Figure 7:**
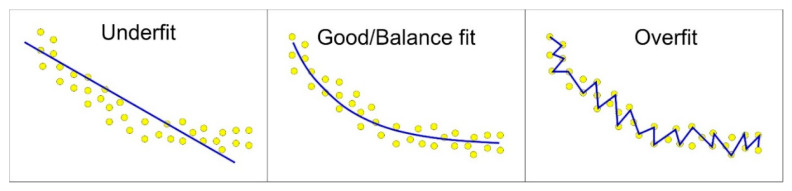
Different types of data fitting.

In order to find the optimal model and structure, different training and transfer functions, listed in Table 3 and Table 4, were trained. The main training parameters were a learning rate of 0.01, a performance target of 0.001 and 1000 epochs. While a neural network is functional and has many potential uses, it has certain limitations. Under or overfitting is one problems that can arise. Overfitting occurs when a network produces a more significant error for a new dataset than for the one it was trained on. Underfitting occurs when the model is too simplistic for training on the chosen dataset. A trained network can remember the acquired knowledge but has not been taught to generalize to unfit data. Figure 7 displays numerous instances of regression data fitting. Since increasing the size of an ensemble set does not reliably improve the accuracy but can decrease the generalizability [72], boosting algorithms are typically accompanied by regularization approaches to prevent overfitting [73]. Using an extensive network to provide a good fit, training multiple networks to ensure good generalization, averaging the outputs of the trained numerous neural networks, randomly separating data and tuning the complexity of a network through regularization [74] are just a few examples of how to improve and handle network generalization. In the MLP network created for the purpose of prediction in this study, generalization was improved by the so-called early stopping method. Early stopping to improve generalization was used in this study’s MLP network for prediction. All supervised network generation functions, including backpropagation networks, use early stopping by default [2]. If data overfitting restarts during network training, validation subset errors will increase. If the validation error rises over the minimum at a significantly different iteration number and exceeds the test subset error, the training stops and network weights and biases return to the minimal validation error [71].

**Figure 8:**
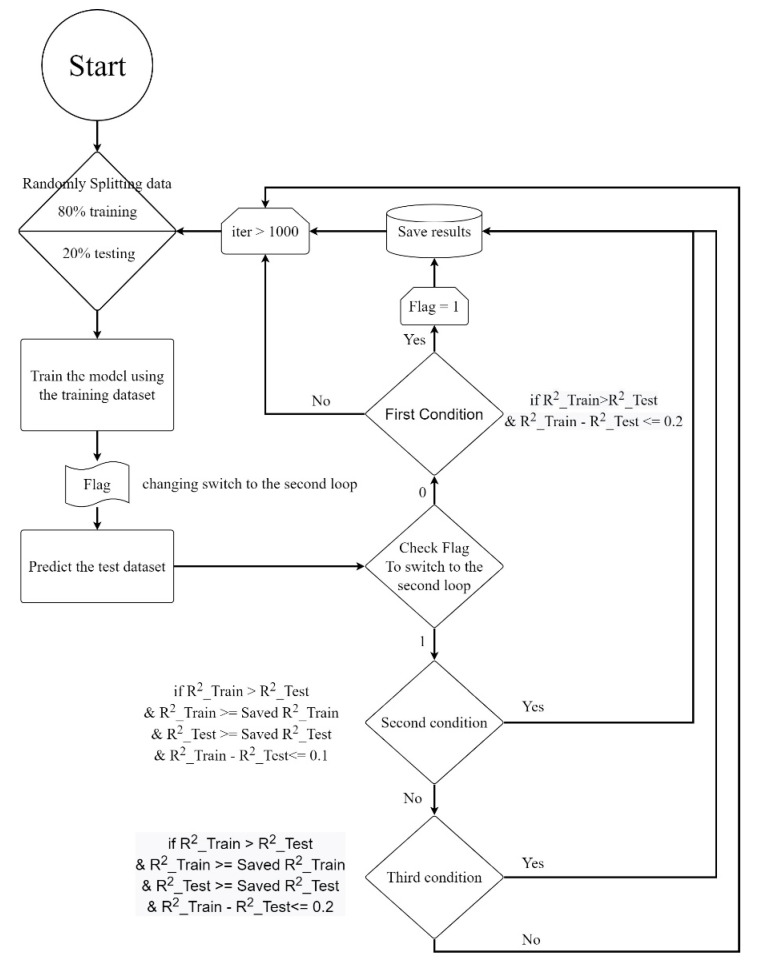
Flowchart of the MLP model that was developed.

In neural networks, dataset training uses optimization to tune and find network weights for a good prediction map. Optimization algorithms are called training functions. Training the network to identify a given input and map it to an output is the training function. Performance goals, trained datasets, weights and biases affect the training function. Selecting a suitable training function for the network is one challenge in making good, rapid and accurate predictions. The transfer function computes each layer’s output by adopting cumulative weights entering the layer. Transfer functions depend on network structure and are difficult to set. This study used fifteen transfer functions individually to increase the prediction accuracy. The output layer’s linear (Purelin) transfer function was chosen in all circumstances. Figure 8 depicts the generated model’s training flowchart as well as the testing procedure utilizing test data. During the running process, two major conditions create decisions. The first loop preserves the model and all variables in the first condition with low conditional limits. After the first condition is met, the second loop is initiated and terminated by a stricter condition. The second loop starts the training and compares them to the variables saved from the prior training and repeats this process until 1000 iterations are completed. The third loop increases the conditional limit in case of not fulfilling the condition requirements in the second loop. To save the results, shared step loops are provided.

Collected data must be divided into training, validation and testing sets. The dataset’s training and testing subsets affect prediction accuracy and training performance [75]. Benchmark performance suffers from inappropriate subsets. Shahin [76] said the dataset’s splitting ratio has no apparent relationship, whereas Zhang et al. [75] reported it as one of the primary issues. However, there is no standard setting for the mentioned issue. Most researchers separate the datasets into lines with varying subgroup ratios based on their surveys. 90% vs. 10%, 80% vs. 20%, or 70% vs. 30% are the most common training and testing ratios. In this paper’s training run, the optimal prediction was achieved by dividing the actual data (72 samples) into training and testing datasets at 80% and 20%, respectively. The dataset consisted of 72 rows from experimental components used for training and testing.

## 3. Results and Discussion

### 3.1. Experimental Results

**Figure 9:**
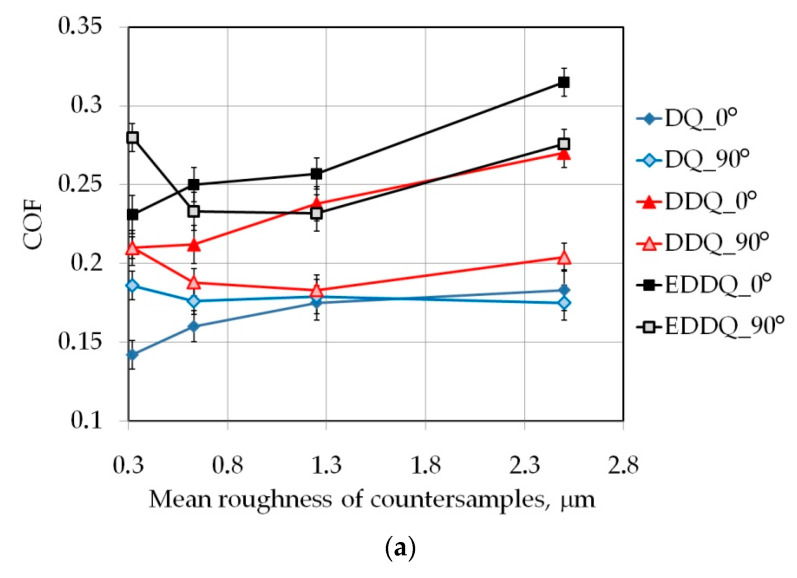

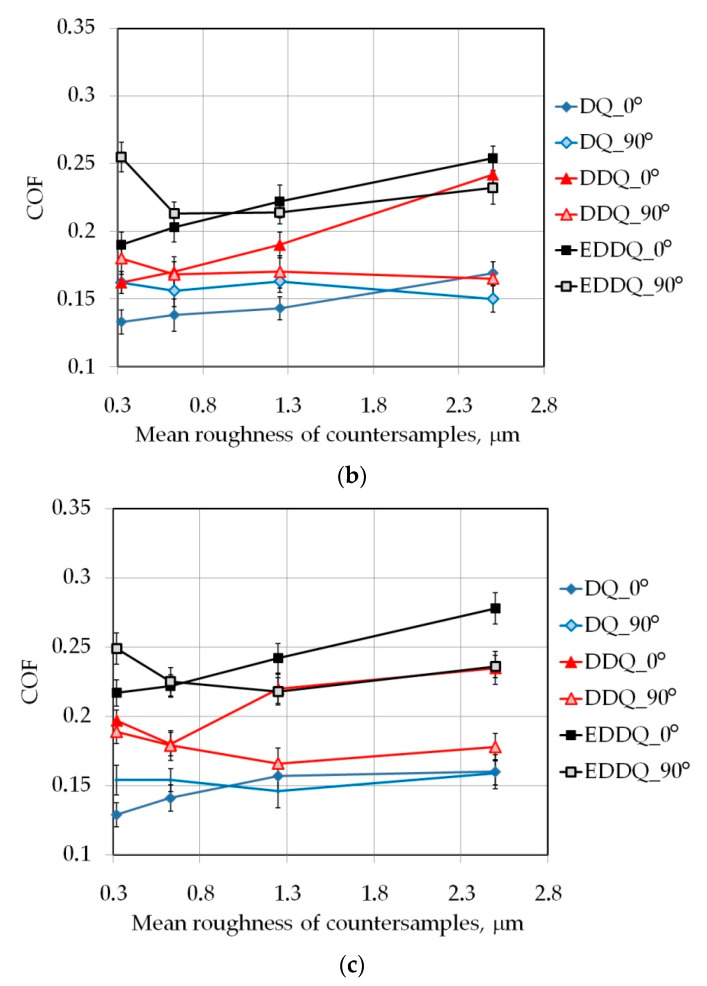
Effect of mean roughness of counter-samples on the COF determined in (**a**) dry friction conditions and lubrication with (**b**) LAN46 machine oil and **(c)** LHL32 hydraulic oil.

In general, the value of the coefficient of friction increased with increasing surface roughness of the counter-samples (Figure 9). It is clear that in dry friction conditions and for the surface roughness of counter-samples Ra = 0.32 μm (Figure 9a), samples cut perpendicularly to the rolling direction of the sheet showed a greater value for the coefficient of friction than samples cut along the sheet rolling direction. With a further increase in the roughness of the counter-samples, the relationship was reversed. This is due to the large contact surface that occurs when friction is made between surfaces with low roughness. Under these conditions, there is a strong mechanical interaction of the surface asperities, which in the case of sheet metal are displaced as a result of the texture created in the cold-rolling process of the sheet metal. A similar situation occurred during friction tests under lubrication with LAN46 machine oil (Figure 9b) and LHL32 hydraulic oil (Figure 9c). The influence of roughness on the effectiveness of lubrication is most important during the friction of bodies with low roughness. Then, it is possible to form valleys creating oil pockets on the friction face in which the hydrostatic pressure of the oil closed in the valleys creates a so-called “lubricant cushion” limiting the metallic contact of the friction faces. As the roughness of the counter-samples increases, the area of contact surface decreases, but a most intensive interaction occurs between the tool roughness asperities and the relatively soft material of the sample.

To investigate the effect of lubricant on the friction coefficient, let us enter the parameter effectiveness of lubrication (EOL) determined according to the formula:

(3) $$\mathrm{EOL}=\frac{\mathrm{COF}\;\left(\mathrm{dry}\;\mathrm{friction}\right)-\mathrm{COF}\left(\mathrm{lubricated}\;\mathrm{conditions}\right)}{\mathrm{COF}\;\left(\mathrm{dry}\;\mathrm{friction}\right)}\cdot 100\%$$

**Figure 10:**
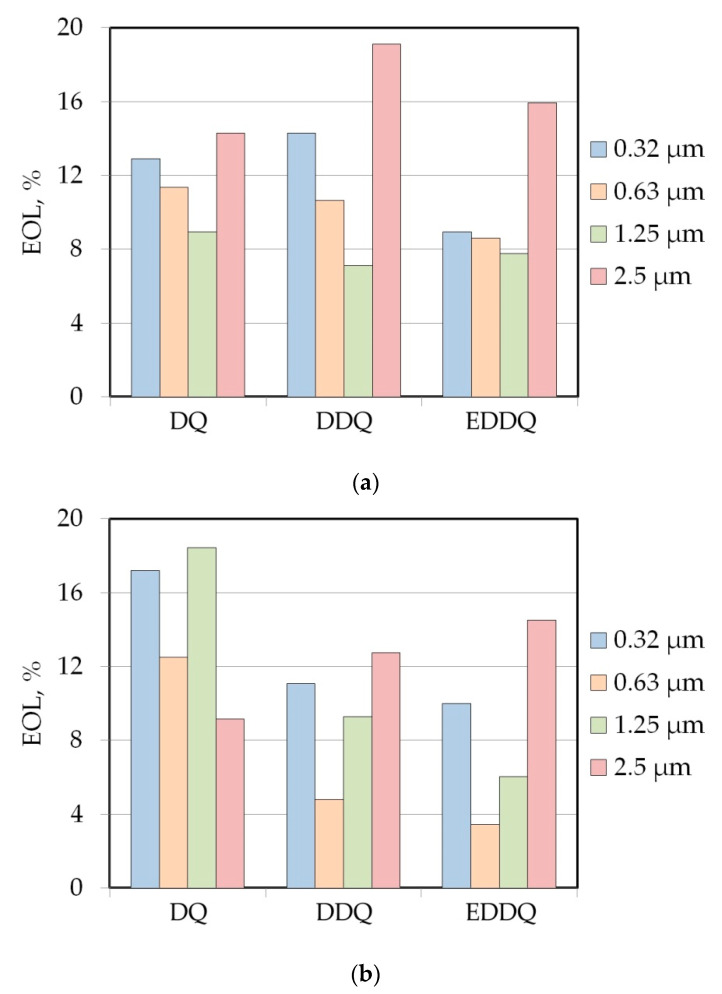
Effectiveness of lubrication for samples cut perpendicular to the sheet rolling direction tested with the presence of (**a**) LAN46 machine oil and (**b**) LHL32 hydraulic oil.

In the case of LAN46 machine oil (Figure 10a), the effectiveness of lubrication decreased in the range of roughness of counter-samples between Ra = 0.32–1.25 μm. It was for the roughness of counter-samples of 2.5 mm that the lubricant decreased the friction coefficient to the greatest extent (14.3–19.1%). The increase in EOL value can be explained by larger spaces that lie between surface asperities of the counter-samples with the parameter Ra = 2.5 μm. In terms of the roughness of the Ra = 0.32–1.25 μm counter-samples, the dominant friction mechanism was mechanical surface flattening and roughening. The influence of the lubricant on COF was less significant under these conditions. The change in viscosity of the lubricant used, i.e., LHL32, significantly changed the influence of the roughness of the counter-samples on the value of the EOL parameter (Figure 10b). 

With increasing drawing quality of the sheet metal, the value of the EOL decreased but only in the range of surface roughness of counter-samples between Ra = 0.32–1.25 μm. In the case of the counter-samples with surface roughness Ra = 2.5 μm, the situation was reversed. Similarly, to lubrication with LAN46 machine oil, the highest lubrication efficiency was observed for counter-samples with a surface roughness Ra = 2.5 μm but only for DDQ and EDDQ materials. Under these conditions, the efficiency of lowering of the friction coefficient by LHL32 hydraulic lubricant (Figure 10b) was generally lower than that of the LAN46 hydraulic oil (Figure 10a). Interpretation of the differences in the efficiency of lubricating oils for the DQ steel sheet was not unambiguous due to many phenomena and parameters that synergistically affect the value of the COF. Therefore, the next section of this article presents the use of artificial neural networks to analyse the experimental results of the DBS test.

### 3.2. Results of ANN Modelling

Using the right validation metric to evaluate a predictive model is crucial. This study compared and validated several structures, training and transferring methods for measuring the agreement between actual and predicted values. Choosing the right validation metric is vital and challenging to evaluate outcomes and reduce errors. All structures and models trained and tested in this work were compared using good metrics to assess test performance. Training and test errors must be distinguished. Test errors are determined using a stored entire dataset unknown to the model, while training errors are derived using the same data. The training dataset R^2^ value reveals variation within the trained samples through the model, while the testing dataset R^2^ value indicates the model’s predictive quality.

MLP’s best outcomes from using several models with varied training and transfer functions have been summarized, with more thorough evaluations shown in Appendix A (Table A1). When material B, SB and SSB were employed, Table 5 shows the best values of numerous validation metrics used to evaluate MLP training and transfer functions performance. Table 5 contains the following statistical parameters: ME—mean error, MAE—mean absolute error, MSE—mean square error, RMSE—root mean square error, MRE—mean relative error, SD—standard deviation, SEM—standard error mean. Models and structures are assessed using R^2^ and adj. R^2^.

**Figure 11:**
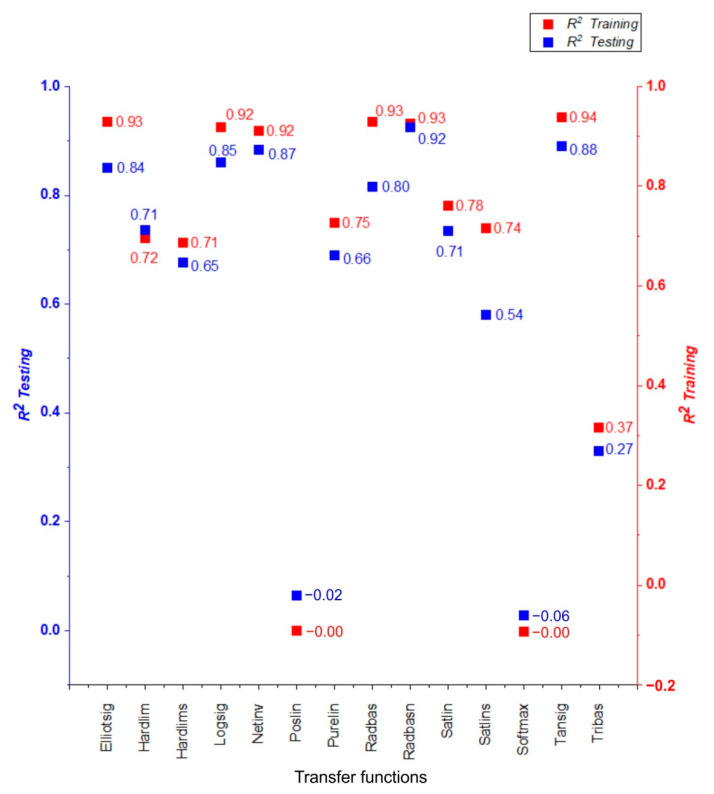
Evaluation of different transfer functions using Bayesian regularization backpropagation (BRB)—Trainbr training function by R^2^ values.

**Figure 12:**
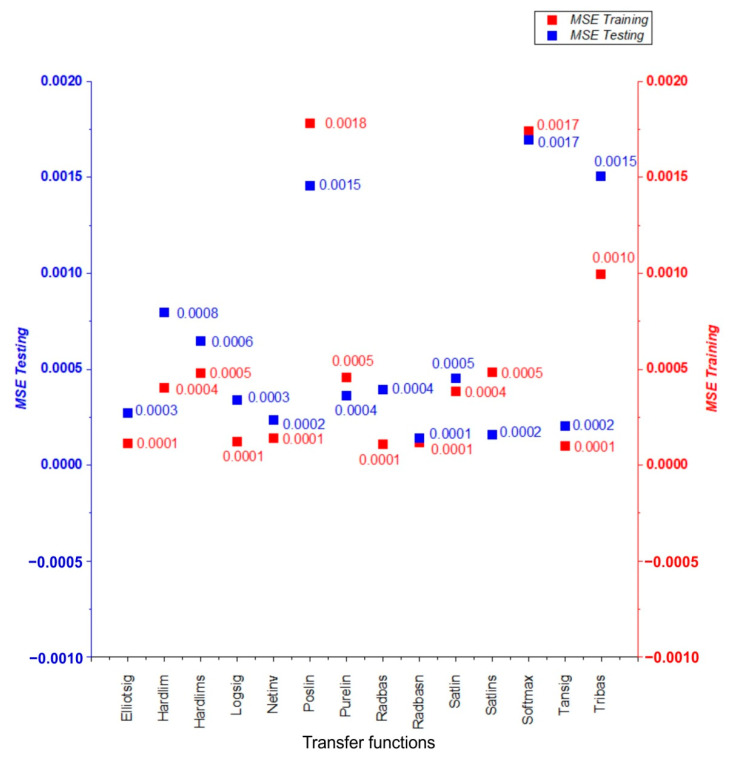
Evaluation of different transfer functions using Bayesian regularization backpropagation (BRB)—Trainbr training function by MSE values.

When the R^2^ of the testing was compared to all algorithms, it was discovered that the constructed MLP model performed the best in terms of COF prediction. Using Bayesian regularization backpropagation (BRB)—Trainbr as a training function and radial basis normalized—Radbasn as a transfer function, the greatest performance in predicting the COF was obtained. See Table A1 in the Appendix A for a summary of the results of all training and transfer functions. Figure 11 and Figure 12 depict R^2^ and MSE, respectively for each of the transfer functions predicted by the ANN using the Trainbr training function.

Instead of constructing, executing and evaluating a new ANN model each time, analytical equations were extracted from the best model to predict COF easily, practically and rapidly. Thus, the Radbasn transfer function is represented by Equation (4). Equation (5) provides a prediction of COF before the weights and biases were included. As a result, Equation (6) demands consistent weights and biases from the best-performing ANN network to predict COF analytically. Equation (7) calculates the COF directly and only by adding the values of the process parameters. The retrieved ANN network weights and biases served as one set of input weights (*IW*) and layer weights (LW). The *IW* is between the inputs and the hidden layer, the LW is between the hidden and output layers, and b1 and b2 are each layer’s biases. The biases *b*1, *b*2, *IW* and *LW* of the best-trained ANN model are shown in Table A2 in Appendix A.

(4) $$f\left(x\right)=radbasn\left(x\right)=\frac{exp^{\left(-x^{2}\right)}}{\sum \left(exp^{\left(-x^{2}\right)}\right)}$$

(5) $$COF_{i}^{predict}=b_{2}+LW\times softmax\left(b_{1}+IW\times x\right)$$

(6) $$COF_{i}^{predict}=b_{2}+LW\times \frac{exp^{{\left(-\left(b_{1}+IW\times x\right)\right)}^{2}}}{\sum^{\;}\left(exp^{{\left(-\left(b_{1}+IW\times x\right)\right)}^{2}}\right)}$$

(7) $$\begin{array}{l} COF_{i}^{predict}\\ =\begin{array}{c} b_{2}\\ \left[0.154802\right] \end{array}\begin{array}{c} LW\\ +\left[\begin{array}{cccccccccc} 0.014177 & 0.004673 & -0.000515 & 0.003907 & 0.000085 & 0.018840 & -0.035940 & -0.002575 & 0.145881 & 0.006270 \end{array}\right] \end{array}\\ \times \frac{{exp}^{{\left(-\left(\begin{array}{c} b_{1}\\ \left[\begin{array}{c} -0.000013\\ 0.000054\\ -0.000036\\ 0.000032\\ -0.000016\\ -0.000073\\ 0.000031\\ 0.000013\\ 0.000001\\ -0.000022 \end{array}\right] \end{array}+\begin{array}{c} IW\\ \left[\begin{array}{cccccccccc} 0.000302 & 0.000674 & 0.000158 & -0.000338 & 0.000167 & 0.000563 & -0.060694 & 0.000182 & -0.044608 & -0.000054\\ 0.000664 & -0.056316 & 0.000150 & -0.004919 & -0.001404 & -0.013884 & 0.058730 & 0.003415 & -0.040035 & -0.004141\\ 0.000043 & 0.000430 & -0.000018 & 0.000113 & -0.000017 & -0.000797 & -0.001589 & 0.000004 & 0.000055 & 0.000174\\ 0.002863 & 0.005001 & -0.001888 & 0.005465 & -0.000267 & -0.032309 & -0.004534 & 0.000139 & 0.007430 & 0.006514\\ -0.002295 & -0.003263 & 0.008779 & 0.000295 & -0.002455 & 0.009952 & -0.014893 & 0.002099 & 0.008765 & 0.012974\\ -0.004105 & -0.006876 & 0.008167 & 0.004110 & -0.004589 & 0.007066 & 0.003317 & 0.003924 & 0.010053 & 0.002716\\ -0.006618 & 0.013543 & 0.002309 & 0.010179 & -0.007722 & 0.003993 & -0.004841 & 0.007039 & -0.001046 & 0.004050\\ -0.000002 & 0.000031 & -0.000019 & 0.000011 & -0.000003 & -0.000026 & 0.000006 & 0.000003 & -0.000037 & -0.000017 \end{array}\right] \end{array}\times \begin{array}{c} x\\ \left[\begin{array}{c} Ra\;of\;counter\;samples\;(\mu m)\\ friction\;conditions:\;dry\;and\;oil\\ kinematic\;viscosity\;m^{2}/s^{-1}\\ strengthening\;coefficient\;(Mpa)\\ strain\;hardening\;exponent\\ sample\;orientation\;\left({}^{\circ}\right)\\ yield\;stress\;R_{p0.2}\;\left(Mpa\right)\\ ultimate\;tensile\;stress\;Rm\;\left(Mpa\right) \end{array}\right] \end{array}\right)\right)}^{2}}}{\sum^{\;}\left({exp}^{{\left(-\left(\begin{array}{c} b_{1}\\ \left[\begin{array}{c} -0.000013\\ 0.000054\\ -0.000036\\ 0.000032\\ -0.000016\\ -0.000073\\ 0.000031\\ 0.000013\\ 0.000001\\ -0.000022 \end{array}\right] \end{array}+\begin{array}{c} IW\\ \left[\begin{array}{cccccccccc} 0.000302 & 0.000674 & 0.000158 & -0.000338 & 0.000167 & 0.000563 & -0.060694 & 0.000182 & -0.044608 & -0.000054\\ 0.000664 & -0.056316 & 0.000150 & -0.004919 & -0.001404 & -0.013884 & 0.058730 & 0.003415 & -0.040035 & -0.004141\\ 0.000043 & 0.000430 & -0.000018 & 0.000113 & -0.000017 & -0.000797 & -0.001589 & 0.000004 & 0.000055 & 0.000174\\ 0.002863 & 0.005001 & -0.001888 & 0.005465 & -0.000267 & -0.032309 & -0.004534 & 0.000139 & 0.007430 & 0.006514\\ -0.002295 & -0.003263 & 0.008779 & 0.000295 & -0.002455 & 0.009952 & -0.014893 & 0.002099 & 0.008765 & 0.012974\\ -0.004105 & -0.006876 & 0.008167 & 0.004110 & -0.004589 & 0.007066 & 0.003317 & 0.003924 & 0.010053 & 0.002716\\ -0.006618 & 0.013543 & 0.002309 & 0.010179 & -0.007722 & 0.003993 & -0.004841 & 0.007039 & -0.001046 & 0.004050\\ -0.000002 & 0.000031 & -0.000019 & 0.000011 & -0.000003 & -0.000026 & 0.000006 & 0.000003 & -0.000037 & -0.000017 \end{array}\right] \end{array}\times \begin{array}{c} x\\ \left[\begin{array}{c} Ra\;of\;counter\;samples\;(\mu m)\\ friction\;conditions:\;dry\;and\;oil\\ kinematic\;viscosity\;m^{2}/s^{-1}\\ strengthening\;coefficient\;(Mpa)\\ strain\;hardening\;exponent\\ sample\;orientation\;\left({}^{\circ}\right)\\ yield\;stress\;R_{p0.2}\;\left(Mpa\right)\\ ultimate\;tensile\;stress\;Rm\;\left(Mpa\right) \end{array}\right] \end{array}\right)\right)}^{2}}\right)} \end{array}$$

**Figure 13:**
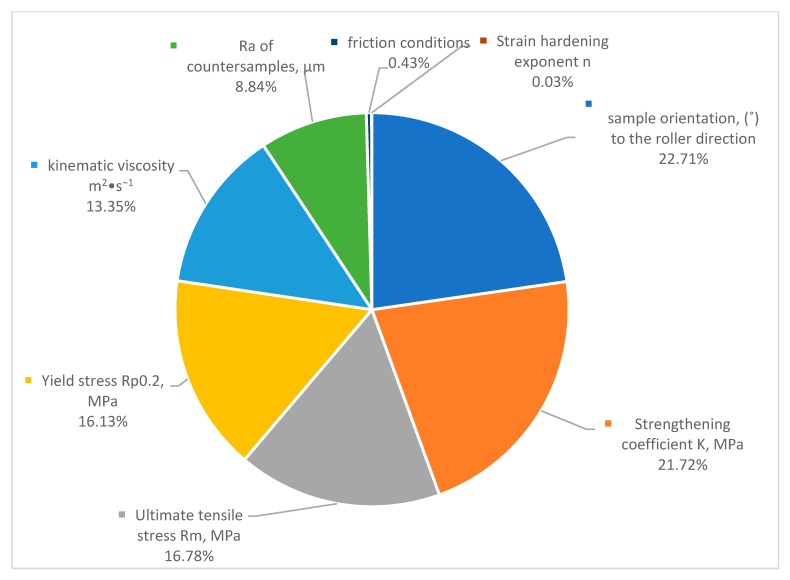
The relative importance and weight analysis of several important factors that affect COF.

Feature, variable or relative importance is the examination of the input variables on the outputs. However, this analysis shows how each feature’s value affects the model’s prediction averages, indicating its relative importance. Substituting the input variables with high relative importance (RI) values affects results more than variables with lower RI values [68,77,78]. Garson [79], most squares [80], and connection weights [81] calculate the feature importance. Various research has calculated and assessed variable impacts using feature importance [78,82,83,84,85,86,87,88]. Figure 13 shows the relative importance and weight analysis of several important factors that affect COF. There are different ways to determine how the input variables affect the output, which is the COF, based on weights and biases. However, the Garson method was adopted in this study. The calculation shows that changes in the sample orientation (°) to the roller direction and strengthening coefficient K (MPa) significantly affect COF. Ultimate tensile stress R_m_ (MPa) and yield stress R_p0_._2_ (MPa) came in second concerning COF. Friction conditions and strain hardening exponent had a minor effect. It is possible that the relative importance regarding the friction conditions is not reasonable because the data has been categorically encoded and binarized using one hot encoding. All the actual and predicted COF values for the training and testing datasets are listed in the Appendix A (Table A3).

## 4. Conclusions

This article presented the results of ANN analysis of the friction behaviour of drawing quality steel sheets in drawbead profiles during sheet metal forming. The experimental results were used to train artificial neural networks. The following conclusions can be drawn from the results presented in this article:

- A trend of increasing the COF with increasing the mean surface roughness of the counter-samples was observed for all friction conditions analysed.
- As the surface roughness of the counter-samples increased, the area of contact surface decreased; however, the resulting coefficient of friction is a result of the coexistence of the following mechanisms: surface flattening and roughening, and lubrication.
- Favourable lubrication conditions occur with low roughness of a soft friction pair (workpiece) compared to the surface properties of the counter-samples.
- The MLP algorithm successfully predicted the coefficient of friction with R^2^ values from 0.9318 for the training dataset and 0.9180 for the testing dataset, with an average R^2^ value of 0.9293 for both the training and testing datasets.
- When it came to predict the coefficient of friction accurately, the Bayesian regularization backpropagation (BRB)—Trainbr training method fared the best. The radial basis normalized—Radbasn transfer function provided the best prediction of the COF.
- The relative significance (RI) methods given that sample orientation to the sheet rolling direction and strengthening coefficient significantly affected COF. The ultimate tensile and yield stress are tied in second place in terms of influences on COF. There is only a slight influence from the friction conditions and the strain hardening exponent.

## Figures and Tables

**Table 1:** Basic mechanical parameters of the sheets tested.

Material	Orientation	Yield Stress R_p0.__2_, MPa	Ultimate Tensile Stress R_m_, MPa	Strengthening Coefficient K, MPa	Strain Hardening Exponent n
DQ	0°	193	351	554	0.166
	90°	193	353	563	0.174
DDQ	0°	196	336	557	0.192
	90°	198	311	526	0.177
EDDQ	0°	151	282	494	0.221
	90°	153	287	487	0.211

**Table 2:** The basic physical properties of oils.

Oil	Kinematic Viscosity, mm^2^/s	Viscosity Index	Density kg/m^3^
LAN46	43.9	94	875
LHL32	32	95	875

**Table 3:** Details of training functions used in multilayer perceptron.

Algorithm	Acronym	Long Term
Trainlm	LM	Levenberg–Marquardt
Trainbfg	BFG	BFGS Quasi-Newton
Trainrp	RP	Resilient Backpropagation
Trainscg	SCG	Scaled Conjugate Gradient
Traincgb	CGB	Conjugate Gradient with Powell/Beale Restarts
Traincgf	CGF	Fletcher–Powell Conjugate Gradient
Traincgp	CGP	Polak–Ribiére Conjugate Gradient
Trainoss	OSS	One Step Secant
Traingdx	GDX	Variable Learning Rate Backpropagation
Trainbr	BRB	Bayesian Regularization Backpropagation

**Table 4:** Details of transfer functions used in multilayer perceptron.

Acronym	Long Term	Equations
Elliotsig	Elliot sigmoid	*f(x) = elliotsig(x) = (x)/1+|x|*
Hardlim	Positive hard limit	*f(x) = hardlim(x) = 1, if x ≥ 0;* *=0, otherwise.*
Hardlims	Symmetric hard limit	*f(x) = hardlim(x) = 1, if x ≥ 0;* *=−1, otherwise.*
Logsig	Logarithmic sigmoid	*f(x) = logsig(x) = 1/(1 + exp(−x))*
Netinv	Inverse	*f(x) = netinv (x) = 1/x*
Poslin	Positive linear	*f(x) = poslin(x) = x, if x* *≥* *0;* *=0, if x ≤ 0.*
Purelin	Linear	*f(x) = purelin(x) = x*
Radbas	Radial basis	*f(x) = radbas(x) = exp (−x^2^)*
Radbasn	Radial basis normalized	*f(x) = radbasn(x) = exp (−x^2^)/sum(exp(−x^2^))*
Satlin	Positive saturating linear	*f(x) = satlin(x) = 0, if x ≤ 0;* *=x, if 0 ≤ x ≤ 1;* *=1, if 1 ≤ x.*
Satlins	Symmetric saturating linear	*f(x) = satlins(x) = −1, if x ≤ −1;* *=x, if −1 ≤ x ≤ 1;* *=1, if 1 ≤ x.*
Softmax	Soft max	*f(x) = softmax(x) = exp(x)/sum(exp(x))*
Tansig	Symmetric sigmoid	*f(x) = tansig(x) = 2/(1+exp (−2* *∗* *x)) − 1*
Tribas	Triangular basis	*f(x) = tribas(x) = 1 − abs(x), if −1 ≤ x ≤ 1;* *=0, otherwise.*

Where *x* is the weighted sum of *w*, *i*, *b*, and *y* of Equation (2).

**Table 5:** Validation metrics of the best multilayer perceptron (MLP) models for predicting COF.

Training Function	Bayesian Regularization Backpropagation (BRB)—Trainbr		
Transfer Function	Radial Basis Normalized—Radbasn		
Validation Metric	Training	Testing	Average (Training and Testing)
ME	0.0005	−0.0014	0.0001
MAE	0.0081	0.0086	0.0082
MSE	0.0001	0.0001	0.0001
RMSE	0.0108	0.0118	0.0110
MRE	0.0404	0.0415	0.0406
SD	0.0109	0.0122	0.0111
SEM	0.0014	0.0033	0.0013
R^2^	0.9318	0.9180	0.9293
adj. R^2^	0.9219	0.9160	0.9195

## Data Availability

Data are contained within the article.

## Appendix A

**Table A1:** The validation metrics of all training and transfer functions of the multilayer perceptron (MLP) models for predicting COF.

Training Function		Levenberg–Marquardt (LM)—Trainlm													
Transfer Function		Elliotsig	Hardlim	Hardlims	Logsig	Netinv	Poslin	Purelin	Radbas	Radbasn	Satlin	Satlins	Softmax	Tansig	Tribas
Training	R^2^	0.8730	0.6622	0.7774	0.3718	0.9315	0.8189	0.8414	0.2051	0.8735	0.8779	0.8775	−0.0008	−0.0182	0.0319
	MSE	0.0002	0.0006	0.0004	0.0010	0.0001	0.0003	0.0002	0.0014	0.0002	0.0002	0.0002	0.0016	0.0017	0.0016
Testing	R^2^	0.7875	0.4633	0.6164	0.2741	0.8913	0.7777	0.8049	0.0418	0.7690	0.8459	0.8527	−0.0044	−0.1474	0.0301
	MSE	0.0004	0.0007	0.0005	0.0016	0.0002	0.0005	0.0006	0.0015	0.0004	0.0004	0.0002	0.0021	0.0022	0.0019
Training function		BFGS Quasi-Newton (BFG)—Trainbfg													
Training	R^2^	0.5293	0.6093	0.6994	−0.0006	0.8169	−0.0045	0.0896	0.0000	−0.0047	−0.0007	−0.0002	0.0103	−0.0043	−0.8322
	MSE	0.0008	0.0007	0.0006	0.0019	0.0003	0.0018	0.0014	0.0018	0.0019	0.0017	0.0018	0.0014	0.0017	0.0032
Testing	R^2^	0.3481	0.5398	0.5154	−0.0518	0.6747	−0.0326	−0.0501	−0.0621	−0.0078	−0.1610	−0.1712	−0.0543	−0.0266	−0.9412
	MSE	0.0009	0.0006	0.0004	0.0009	0.0004	0.0014	0.0026	0.0014	0.0010	0.0017	0.0016	0.0032	0.0021	0.0031
Training function		Resilient Backpropagation (RP)—Trainrp													
Training	R^2^	0.8856	0.5644	0.7729	0.8950	0.8701	−0.2627	0.5016	−0.0036	−0.0307	−0.0206	−0.0076	−0.5355	0.0000	−0.6498
	MSE	0.0002	0.0007	0.0004	0.0002	0.0002	0.0022	0.0009	0.0016	0.0017	0.0015	0.0017	0.0029	0.0018	0.0031
Testing	R2	0.7544	0.4247	0.7373	0.8830	0.6753	−0.3073	0.4009	−0.0046	−0.2294	−0.1607	−0.0947	−0.5593	−0.0197	−0.7014
	MSE	0.0003	0.0010	0.0006	0.0002	0.0008	0.0019	0.0007	0.0022	0.0022	0.0032	0.0019	0.0013	0.0012	0.0017
Training function		Scaled Conjugate Gradient (SCG)—Trainscg													
Training	R^2^	0.8965	0.7040	0.7380	0.9293	0.7203	−2.8003	0.6393	0.0589	0.4909	−0.0007	0.0412	−0.0009	0.5936	−4.9884
	MSE	0.0002	0.0005	0.0005	0.0001	0.0005	0.0062	0.0006	0.0016	0.0007	0.0016	0.0017	0.0018	0.0007	0.0100
Testing	R^2^	0.7557	0.5815	0.5412	0.9235	0.6609	−2.9296	0.6249	−0.0775	0.4900	−0.0302	−0.1379	−0.0308	0.5858	−5.0740
	MSE	0.0006	0.0006	0.0008	0.0002	0.0006	0.0077	0.0006	0.0019	0.0014	0.0021	0.0018	0.0013	0.0006	0.0101
Training function		Conjugate Gradient with Powell/Beale Restarts (CGB)—Traincgb													
Training	R^2^	0.9111	0.6653	0.7823	0.8965	−0.0214	0.5384	0.6662	−0.0001	−0.0059	0.7371	−0.0005	−0.0261	0.6954	0.0000
	MSE	0.0002	0.0006	0.0004	0.0002	0.0018	0.0008	0.0006	0.0016	0.0015	0.0004	0.0018	0.0019	0.0005	0.0017
Testing	R^2^	0.8293	0.5424	0.6019	0.7815	−0.0428	0.4136	0.4838	−0.0602	−0.0497	0.5875	−0.0083	−0.0858	0.6272	−0.0289
	MSE	0.0003	0.0006	0.0006	0.0003	0.0016	0.0008	0.0009	0.0021	0.0026	0.0010	0.0016	0.0014	0.0007	0.0018
Training function		Fletcher–Powell Conjugate Gradient (CGF)—Traincgf													
Training	R^2^	0.8495	0.5491	0.5807	0.5103	0.3220	−1.6810	0.6662	−0.0015	−0.0002	−0.0002	−0.0031	−0.0014	−0.0020	−0.0001
	MSE	0.0003	0.0007	0.0007	0.0008	0.0012	0.0046	0.0006	0.0017	0.0018	0.0019	0.0018	0.0017	0.0019	0.0017
Testing	R^2^	0.7948	0.5297	0.5495	0.3660	0.1933	−1.8698	0.4838	−0.0412	−0.0221	−0.0142	−0.0271	−0.0056	−0.0089	−0.1788
	MSE	0.0004	0.0010	0.0007	0.0013	0.0012	0.0048	0.0009	0.0017	0.0014	0.0011	0.0014	0.0018	0.0010	0.0018
Training function		Polak–Ribiére Conjugate Gradient (CGP)—Traincgp													
Training	R^2^	0.5955	0.5645	0.7784	0.0086	0.3507	−4.4318	−77.6014	−0.0010	−0.0005	−0.0016	−0.0029	0.0000	0.4306	−0.0003
	MSE	0.0007	0.0007	0.0003	0.0017	0.0011	0.0099	0.1404	0.0018	0.0017	0.0014	0.0019	0.0019	0.0010	0.0014
Testing	R^2^	0.4401	0.4315	0.7301	−0.0084	0.2503	−4.5725	−77.7437	−0.0942	−0.0848	−0.0720	−0.0625	−0.1148	0.2817	−0.0188
	MSE	0.0009	0.0009	0.0006	0.0016	0.0014	0.0073	0.0937	0.0013	0.0020	0.0029	0.0010	0.0010	0.0011	0.0029
Training function		One Step Secant (OSS)—Trainoss													
Training	R^2^	0.5604	−0.0399	−0.1893	−0.0036	0.5851	0.2590	0.2152	−0.3388	−0.0002	−0.0002	−0.0003	0.0000	−0.0001	0.0000
	MSE	0.0007	0.0017	0.0020	0.0017	0.0008	0.0013	0.0013	0.0024	0.0017	0.0019	0.0020	0.0017	0.0019	0.0017
Testing	R^2^	0.5409	−0.1051	−0.2987	−0.0484	0.4091	0.2245	0.1251	−0.3659	−0.0188	−0.0142	−0.0160	−0.0117	−0.0156	−0.0176
	MSE	0.0006	0.0021	0.0022	0.0016	0.0008	0.0010	0.0016	0.0021	0.0016	0.0011	0.0007	0.0018	0.0010	0.0018
Training function		Variable Learning Rate Backpropagation (GDX)—Traingdx													
Training	R^2^	−0.0005	0.7337	0.6988	−1.4754	0.3543	−0.0001	0.6662	−0.3164	−0.0014	−0.0011	0.4030	0.0000	−0.0006	−0.0022
	MSE	0.0017	0.0005	0.0005	0.0046	0.0011	0.0015	0.0006	0.0023	0.0018	0.0017	0.0010	0.0017	0.0016	0.0015
Testing	R^2^	−0.0009	0.6728	0.5573	−1.6742	0.3401	−0.1135	0.4838	−0.4212	−0.0509	−0.0116	0.3546	−0.0383	−0.1115	−0.0801
	MSE	0.0017	0.0005	0.0008	0.0031	0.0011	0.0028	0.0009	0.0023	0.0013	0.0019	0.0013	0.0017	0.0024	0.0027
Training function		Bayesian Regularization Backpropagation (BRB)—Trainbr													
Training	R^2^	0.9344	0.7203	0.7128	0.9242	0.9188	−0.0011	0.7484	0.9340	0.9318	0.7811	0.7384	−0.0024	0.9435	0.3717
	MSE	0.0001	0.0004	0.0005	0.0001	0.0001	0.0018	0.0005	0.0001	0.0001	0.0004	0.0005	0.0017	0.0001	0.0010
Testing	R^2^	0.8360	0.7120	0.6457	0.8475	0.8728	−0.0208	0.6610	0.7986	0.9180	0.7102	0.5419	−0.0619	0.8796	0.2674
	MSE	0.0003	0.0008	0.0006	0.0003	0.0002	0.0015	0.0004	0.0004	0.0001	0.0005	0.0002	0.0017	0.0002	0.0015

**Table A2:** The weights and biases of the best MLP model for predicting COF (Trainbr with Radbasn).

*b*_1_	*b*_2_	*IW*								*LW*
−0.000013	0.154802302	0.000302	0.000664	0.000043	0.002863	−0.002295	−0.004105	−0.006618	−0.000002	0.014177
0.000054		0.000674	−0.056316	0.000430	0.005001	−0.003263	−0.006876	0.013543	0.000031	0.004673
−0.000036		0.000158	0.000150	−0.000018	−0.001888	0.008779	0.008167	0.002309	−0.000019	−0.000515
0.000032		−0.000338	−0.004919	0.000113	0.005465	0.000295	0.004110	0.010179	0.000011	0.003907
−0.000016		0.000167	−0.001404	−0.000017	−0.000267	−0.002455	−0.004589	−0.007722	−0.000003	0.000085
−0.000073		0.000563	−0.013884	−0.000797	−0.032309	0.009952	0.007066	0.003993	−0.000026	0.018840
0.000031		−0.060694	0.058730	−0.001589	−0.004534	−0.014893	0.003317	−0.004841	0.000006	−0.035940
0.000013		0.000182	0.003415	0.000004	0.000139	0.002099	0.003924	0.007039	0.000003	−0.002575
0.000000		−0.044608	−0.040035	0.000055	0.007430	0.008765	0.010053	−0.001046	−0.000037	0.145881
−0.000022		−0.000054	−0.004141	0.000174	0.006514	0.012974	0.002716	0.004050	−0.000017	0.006270

**Table A3:** All the actual and predicted values of COF (trained and tested sets).

Training Dataset			Testing Dataset		
	Actual	Predicted		Actual	Predicted
1	0.183	0.188	1	0.236	0.235
2	0.188	0.198	2	0.156	0.157
3	0.257	0.265	3	0.178	0.179
4	0.217	0.211	4	0.175	0.178
5	0.162	0.157	5	0.233	0.251
6	0.183	0.197	6	0.133	0.137
7	0.168	0.172	7	0.203	0.204
8	0.17	0.172	8	0.27	0.267
9	0.162	0.166	9	0.19	0.194
10	0.278	0.275	10	0.18	0.171
11	0.16	0.168	11	0.28	0.252
12	0.154	0.161	12	0.166	0.178
13	0.22	0.207	13	0.16	0.153
14	0.165	0.173	14	0.25	0.250
15	0.186	0.177	15	0.197	0.176
16	0.235	0.244	16	0.179	0.178
17	0.157	0.149	17	0.212	0.218
18	0.222	0.223	18	0.236	0.235
19	0.231	0.240			
20	0.142	0.148			
21	0.222	0.223			
22	0.232	0.250			
23	0.129	0.139			
24	0.238	0.238			
25	0.214	0.230			
26	0.225	0.237			
27	0.19	0.195			
28	0.213	0.230			
29	0.265	0.231			
30	0.249	0.237			
31	0.146	0.162			
32	0.189	0.178			
33	0.179	0.178			
34	0.159	0.163			
35	0.175	0.163			
36	0.232	0.229			
37	0.169	0.162			
38	0.21	0.198			
39	0.176	0.177			
40	0.18	0.186			
41	0.204	0.197			
42	0.138	0.140			
43	0.315	0.285			
44	0.254	0.255			
45	0.154	0.161			
46	0.218	0.236			
47	0.242	0.244			
48	0.141	0.142			
49	0.276	0.248			
50	0.17	0.175			
51	0.21	0.207			
52	0.143	0.146			
53	0.15	0.158			
54	0.242	0.233			
55	0.163	0.157			
56	0.236	0.235			
